# NF-*κ*B-Activated lncRNACASC9 Promotes Bladder Cancer Progression by Regulating the TK1 Expression

**DOI:** 10.1155/2022/9905776

**Published:** 2022-05-07

**Authors:** Cai Longjun, Zhang Jianjun, Pang Kun, Hao Lin, Shi Zhenduo, Dong Yang, Li Bibo, Zhang Zhiguo, Li Rui, Han Conghui

**Affiliations:** ^1^Medical College of Soochow University, Suzhou, 215123 Jiangsu, China; ^2^Department of Urology, The Affiliated Suqian Hospital of Xuzhou Medical University, Suqian People's Hospital of Nanjing Drum-Tower Hospital Group, Suqian, 223800 Jiangsu, China; ^3^Department of Urology, Xuzhou Central Hospital, Xuzhou, 221009 Jiangsu, China; ^4^Department of Urology, Taizhou Traditional Chinese Medicine Hospital, Taizhou, 225300 Jiangsu, China; ^5^Department of Central Laboratory, Xuzhou Central Hospital, Xuzhou, 221009 Jiangsu Province, China; ^6^Jiangsu Normal University College, Xuzhou, Jiangsu, China

## Abstract

Long noncoding RNAs (lncRNA) are involved in cancer development, but the roles of most lncRNAs are undocumented. In this study, we identified lncRNAs that were abnormally expressed in bladder cancer. We found that lncRNACASC9 plays an important role in the progression of bladder cancer. CASC9 was highly expressed in bladder cancer cells and tissues, and the prognosis of bladder cancer patients with high expression of CASC9 was poor. The results of colony formation assays, CCK-8 assays, EdU assays, transwell assays, mouse xenograft models, and tail vein injection lung metastasis model showed that CASC9 could promote bladder cancer cells growth and metastasis both in vitro and in vivo. Mechanistically, through FISH experiments, luciferase reporter experiments, and RIP experiments, we proved that CASC9 regulated the expression of TK1 by adsorbing miR-195-5p, thereby exerting an oncogenic effect in bladder cancer. Taken together, our findings support that the CASC9/miR-195-5p/TK1 axis is a critical pathway in the tumorigenesis and progression of bladder cancer, implicating a new therapeutic direction for the treatment of bladder cancer.

## 1. Introduction

Bladder cancer (BCa) is one of the most common malignancies of the urinary tract with more than 4.3 × 10^5^ new cases per year [1]. BCa cancer is classified into two types including nonmuscle invasive and muscle invasive BCa, with the 5-year overall survival(OS) rate of 90% and 60%, respectively [2]. Although great progress has been achieved in the treatment of BCa, the prognosis of BCa remains poor. Therefore, exploring and understanding the molecular mechanism of BCa would help identifying promising therapeutic targets.

Mammalian genomes are widely transcribed, with about 2% of the genomic output encodes for peptides or proteins [3, 4]. The long noncoding RNAs (lncRNAs) are a subset of noncoding RNAs that were reported to be involved in the initiation and progression of various malignancies [5–7].

Cancer susceptibility candidate 9 (CASC9) plays a critical role in varieties of human cancers. For example, CASC9 was upregulated in non-small-cell lung carcinoma (NSCLC) and sponged with miR-130b-3p to regulate ZEB2, thus promoted the progression of NSCLC [8]. Similarly, the oncogenic role of CASC9 was detected in oral squamous cell carcinoma and esophageal squamous cell carcinoma [9, 10]. However, the mechanism and function of CASC9 in BCa progression are largely elusive.

In this work, we found that CASC9 was upregulated in BCa tissues and cell lines. We explored the functional role of CASC9 in BCa progression via altering CASC9 expression levels both in vitro and in vivo. Moreover, the upstream and downstream regulation of CASC9 was detected in vitro. Our work showed that the overexpression of CASC9 is induced by NF-ĸB, and the overexpression of CASC9 promoted the progression of BCa by sponging miR-195-5p to upregulate thymidine kinase 1(TK1) in BCa.

## 2. Materials and Methods

### 2.1. Tissue Samples and Clinical Data Collection

Tumor specimens and paired noncancerous tissues were collected from 30 patients with BCa who underwent resection at the Suqian People's Hospital between July 2017 and July 2019. No patients received anticancer treatment before surgery. Tissue samples were snap-frozen in liquid nitrogen and stored at -80°C until RNA analysis. Written informed consent for research was given by all the patients. This study was approved by the Ethics Committee of Xuzhou Medical University.

### 2.2. Cell Culture

The human BCa cell lines (HT-1197, HT-1376, T24, 5637 and SW780) were purchased from Cell Resource Center of the Shanghai Institute for Biological Sciences, Chinese Academy of Sciences, China. The normal bladder epithelial cell line SV-HUC-1 was purchased from the ATCC, USA. These cells were cultured at 37°C with 5% CO_2_ according to the standard protocols. HT-1197, HT-1376, T24, and 5637 cells were cultured in RPMI 1640 medium supplemented with 10% fetal bovine serum and antibiotics (penicillin 100 U/mL, streptomycin 0.1 mg/mL). SW780 and SV-HUC-1 cells were cultured in DMEM medium with 10% FBS and antibiotics.

### 2.3. RNA Isolation and Quantitative Real-Time PCR

Total RNA was extracted from cultured cells and fresh tissues with TRIzol Reagent (Ambion, USA) according to the manufacturer's protocol. miRNA cDNA was synthesized using a miRNA cDNA Synthesis Kit (CoWin Biosciences, USA). LncRNA and mRNA cDNA were synthesized using a PrimeScriptTM RT Master Mix Kit (TaKaRa, Osaka, Japan). Quantitative real-time PCR (qRT-PCR) was performed using a standard protocol from the SYBR Green PCR kit (Toyobo, Osaka, Japan). The following primer sequences were used for qRT-PCR: for CASC9, TTGGTCAGCCACATTCATGGT (forward) and AGTGCCAATGACTCTCCAGC (reverse); for TK1, GCCAAAGACACTCGCTACAG (forward) and CCCCTCGTCGATGCCTATG (reverse); for NF-*κ*B, GGTGCGGCTCATGTTTACAG (forward) and GATGGCGTCTGATACCACGG (reverse); for GAPDH, GGGAGCCAAAAGGGTCATCA (forward) and TGATGGCATGGACTGTGGTC (reverse).

### 2.4. Plasmid Construction and Cell Transfection

The full-length sequences of NF-*κ*B (NM_001165412.2) and CASC9 (NR_103848.1) were cloned into pcDNA3.1(+) expression vector. The small hairpin RNA targeting CASC9 was synthesized and cloned into the pLKO.1 vector (Sigma, USA). All the plasmids were validated by sequencing. CASC9, NF-*κ*B, and TK1 siRNAs were purchased from the Ambion (USA). MiR-195-5p mimics and inhibitors were synthesized by the Ribobio (China). The plasmid vectors and siRNAs were transfected into BC cells using Lipofectamine 3000 (Invitrogen, USA) as per the manufacturer's protocol. For generation of CASC9-depleted 5637 cell lines, the 5637 cells were transfected with control shRNA (sh NC) or CASC9-targeting shRNA (sh CASC9) and selected in the presence of 2 *μ*g/mL puromycin.

### 2.5. Chromatin Immunoprecipitation (CHIP) Assay

The CHIP assay was performed using the Magna CHIP Kit (Merk-Millipore, USA) following the manufacturer's instructions. Briefly, cells were fixed in 1% formaldehyde solution for 20 min and added glycine to a final concentration of 125 mM with shaking for 5 min. DNA fragments ranging from 200 to 500 bp were obtained by ultrasonication. CHIP experiments were performed with anti-NF-*κ*B (#8242, Cell Signaling Technology, USA) or isotype IgG antibodies. There is CHIP primer for the CASC9 promoter: TTCCACCCTCCATCCCGTGT (forward) and TGAATTCTACCCCCGCCCCA (reverse).

### 2.6. EdU Assay

After transfection, BC cells were seeded into 24-well plates (0.5 × 105 cells/well) and cultured for 48 h. Then, cells were incubated with 10 *μ*M EdU reagent (RiboBio, China) for 2 h at 37°C. Nuclei were stained with Hoechst 33342 (RiboBio, China) for 30 min. The stained cells were photographed under an inverted fluorescent microscope. The percentage of EdU-positive cells was determined.

### 2.7. Transwell Assay

Transwell chambers (Corning Costar, USA) were used to measure cell migration and invasion ability. Briefly, BCa cells were seeded into the upper chambers (precoated matrigel for invasion assay) with 100 *μ*L of serum-free RPMI 1640 medium (5 × 10^4^ for migration, 1 × 10^5^ for invasion), which had been inserted into wells of the 24-well plates containing 10% FBS RPMI 1640 medium. After 24 h, the cells were fixed with 4% paraformaldehyde for 30 min and stained with 0.1% crystal violet for 30 min. The migrated cells in the upper chambers were photographed under a phase-contrast microscope and counted.

### 2.8. Western Blot

Western blotting was performed according to protocol. Briefly, cells were lysed in RIPA buffer. Cellular proteins were collected and subjected to 10% SDS-PAGE and transferred onto PVDF membranes. The membranes were then incubated with specific primary antibodies (anti-cyclin B1 (#12231, Cell Signaling Technology, USA), anti-cyclin D1(#55506, Cell Signaling Technology, USA), anti-cyclin E1 (#20808, Cell Signaling Technology, USA), anti-N-cadherin (#13116, Cell Signaling Technology, USA), anti-vimentin (#5741, Cell Signaling Technology, USA), anti-TK1 (#28755, Cell Signaling Technology, USA), and anti-GAPDH (#2118, Cell Signaling Technology, USA)) overnight at 4°C. After the membranes were incubated with secondary antibodies, they were subjected to immunoblot analysis using an ECL immunoblotting kit according to the manufacturer's protocol.

### 2.9. RNA Fluorescence In Situ Hybridization

Cy3-labeled CASC9 probes were designed and synthesized by Servicebio (Wuhan, China). FISH assays were performed using Fluorescent In Situ Hybridization Kit (RiboBio, China) according to the protocol.

### 2.10. Subcellular Fractionation Location

The separation of the nuclear and cytosolic fractions was performed using the PARIS Kit (Invitrogen, USA) according to the manufacturer's instructions.

### 2.11. RNA Pull-Down Assay

The biotinylated RNA pull-down kit (BersinBio) was used for the RNA pull-down experiment. A total of 10^7^ 5637 cells were washed with PBS and crosslinked by ultraviolet irradiation at 254 nm. Cells were lysed with 1 mL lysis buffer. Biotinylated antisense probe of CASC9 (0.2 nmol) was denatured at 65°C for 10 min and hybridized in lysis buffer at room temperature for 2 h before adding 200 *μ*l streptavidin-coated magnetic beads. Nonspecifically bound RNAs were removed by washing, and bound miRNAs were evaluated by qPCR analysis.

### 2.12. Dual Luciferase Reporter Assay

The wild-type (wt CASC9 and wt TK1-3′UTR) or mutant (mt CASC9 and wt TK1-3′UTR) fragments potentially binding to miR-195-5p were subcloned into pmirGLO (Promega). 1 × 10^5^ of BC cells were seeded in 24-well plates for 24 h. Mimics or inhibitors of miR-195-5p were cotransfected with 2 *μ*g reporter. 24 hours after transfection, dual-luciferase reporter assay (Promega, USA) was performed to measure the relative luciferase activity.

### 2.13. Animal Experiments

For the xenograft tumor model, 5 × 10^6^ 5637 cells (sh NC and sh CASC9 stably transfected) in 0.2 mL PBS were subcutaneously injected into BABL/c nude mice (4-week-old male). The tumor volumes were measured every five days calculated with the following equation: *V* = 0.5 × (length × width^2^). After one month later, the mice were sacrificed, and tumors were surgically dissected. For the tail vein injection lung metastasis model, 1 × 10^6^ 5637 cells (sh NC and sh CASC9 stably transfected) were tail-vein injected into four-week-old male BALB/c nude mice. Two months later, all mice were killed, and the lungs were surgically removed and fixed in 10% neutral phosphate-buffered formalin, followed by HE staining and metastatic nodules analysis.

### 2.14. Statistical Analysis

Statistical analyses were performed using SPSS 22.0 (IBM, USA), and figures were produced using GraphPad Prism 6.0. Differences between the different groups were tested using Student's *t*-test or one-way ANOVA. The Kaplan–Meier method was used to evaluate the survival rate and analyzed by log-rank test. All experimental data were presented as the mean ± S.D. of at least three independent experiments. The correlations were analyzed using Pearson's correlation coefficients. *P* < 0.05 was considered statistically significant.

## 3. Results

### 3.1. CASC9 Is Highly Expressed in Bladder Cancer and Is Related to the Prognosis of Patients

**Figure 1:**
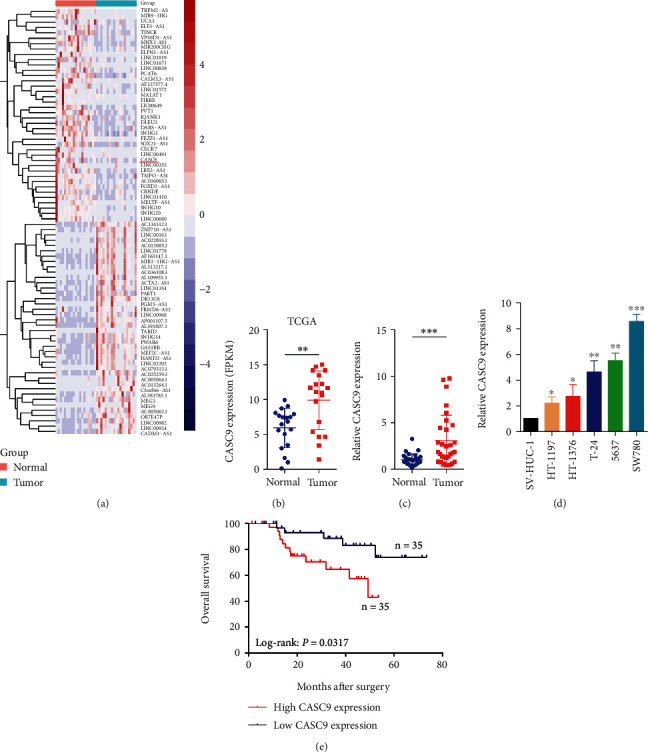
The CASC9 expression is increased in BC, and its expression is related to the prognosis of BC patients. (a) Heatmap of abnormally expressed lncRNAs in bladder cancer. Red in the heat map indicates upregulation, and blue indicates downregulation. The red underline denotes CASC9. (b) The expression of CASC9 in BC generated from sequencing data in the TCGA database. (c) The qRT-PCR analysis of the CASC9 expression in 30 pairs of BC and corresponding adjacent normal tissues. (d)The CASC9 expression in BC cell lines (HT-1197, HT-1376, T24, 5637, and SW780) and normal bladder epithelial cell SV-HUC-1 detected by qRT-PCR. (e) Kaplan-Meier survival analysis of BC patients' overall survival based on the CASC9 expression (*n* = 70, *P* = 0.0317). ^∗^*P* < 0.05, ^∗∗^*P* < 0.01, and ^∗∗∗^*P* < 0.001.

First, we analyzed the transcriptome sequencing data of BCa tissues to determine the abnormally expressed lncRNAs in BCa (Figure 1(a)). Sequencing results showed that CASC9 was significantly overexpressed in BCa tissues (Figure 1(b)). The PCR results of 80 pairs of BCa and adjacent tissues also showed that the expression of CASC9 in tumor tissues was significantly higher than that in adjacent normal tissues (Figure 1(c)). In addition, we detected the expression of CASC9 in the normal bladder epithelial cell line SV-HUC-1 and five BCa cell lines (HT-1197, HT-1376, T24, 5637, and SW780). The results showed that the expression of CASC9 in BCa cell lines was significantly higher than that of normal cells (Figure 1(d)). Besides, survival analysis showed that BCa patients in the CASC9 overexpression group had a worse prognosis (Figure 1(e)). Based on these results, we selected CASC9 for further research.

### 3.2. Transcription Factor NF-*κ*B Activates CASC9 Transcription in Bladder Cancer

**Figure 2:**
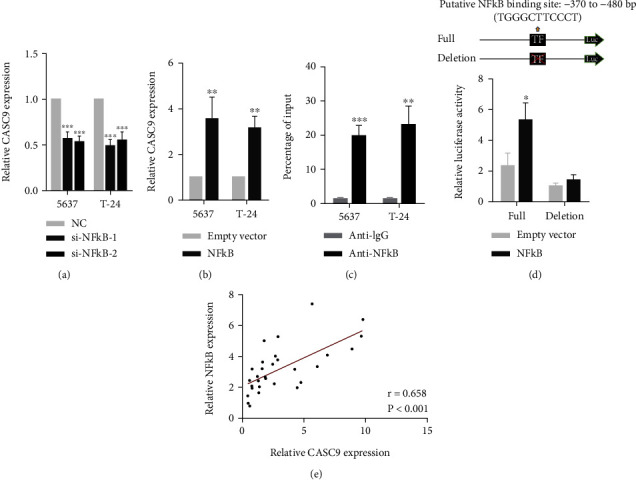
NF-*κ*B activates CASC9 transcription in BC. (a) The CASC9 expression was detected in 5637 and T24 cells transfected with NF-*κ*B siRNAs by qRT-PCR. (b) The CASC9 expression was detected in 5637 and T24 cells transfected with the NF-*κ*B overexpression vector by qRT-PCR. (c) CHIP assays were employed to identify NF-*κ*B occupancy in the CASC9 promoter region. (d) Luciferase reporter assays were conducted to determine the NF-*κ*B binding sites on the CASC9 promoter region. (e) The correlation analysis between NF-*κ*B and CASC9 expression in 30 paired BC samples (*n* = 30, *r* = 0.658, *P* < 0.001). ^∗^*P* < 0.05, ^∗∗^*P* < 0.01, and ^∗∗∗^*P* < 0.001.

Next, we explored why the CASC9 expression is elevated in BCa. We used JASPAR software to predict transcription factors that might bind to the CASC9 promoter region. Among these transcription factors, the prooncogenic transcription factor NF-*κ*B obtained a high score. After silencing NF-*κ*B in BCa cell lines 5637 and T24, the expression of CASC9 was significantly reduced, and after the overexpression of NF-*κ*B, the expression of CASC9 was significantly increased (Figures 2(a) and 2(b)). CHIP experiments with the NF-*κ*B antibody also showed that NF-*κ*B could directly bind to the promoter region of CASC9 (Figure 2(c)). In addition, we designed wild-type and mutant (binding site knockout) promoter luciferase reporter based on the predicted binding sites of NF-*κ*B and CASC9 promoter regions. The results showed that when NF-*κ*B was overexpressed, the wild-type reporter could be activated, but the luciferase activity of the mutant reporter was not significantly different from that of the control group (Figure 2(d)). Besides, correlation analysis indicated that the expression of NF-*κ*B and CASC9 in BCa tissue was significantly positively correlated (Figure 2(e)).

### 3.3. CASC9 Promotes the Growth and Metastasis of Bladder Cancer Cells

**Figure 3:**
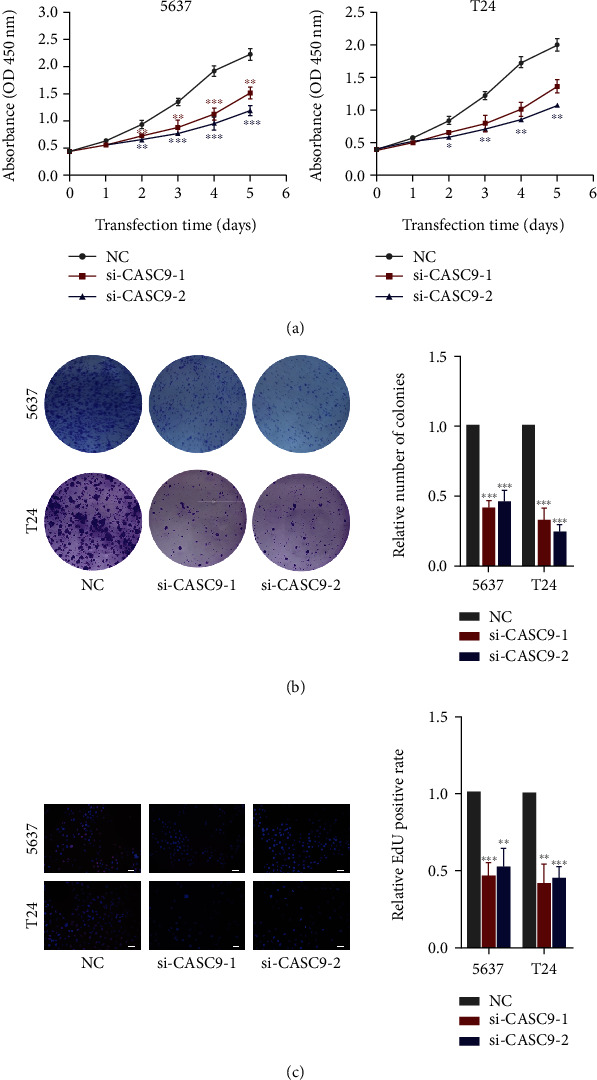
CASC9 promotes BC cells proliferation in vitro. (a) CCK-8 assays of 5637 and T24 cells transfected with CASC9 siRNAs. (b) Colony formation assays of 5637 and T24 cells transfected with CASC9 siRNAs. (c) EdU assays of 5637 and T24 cells transfected with CASC9 siRNAs. Scar bar = 50 *μ*m, ^∗^*P* < 0.05, ^∗∗^*P* < 0.01, and ^∗∗∗^*P* < 0.001.

**Figure 4:**
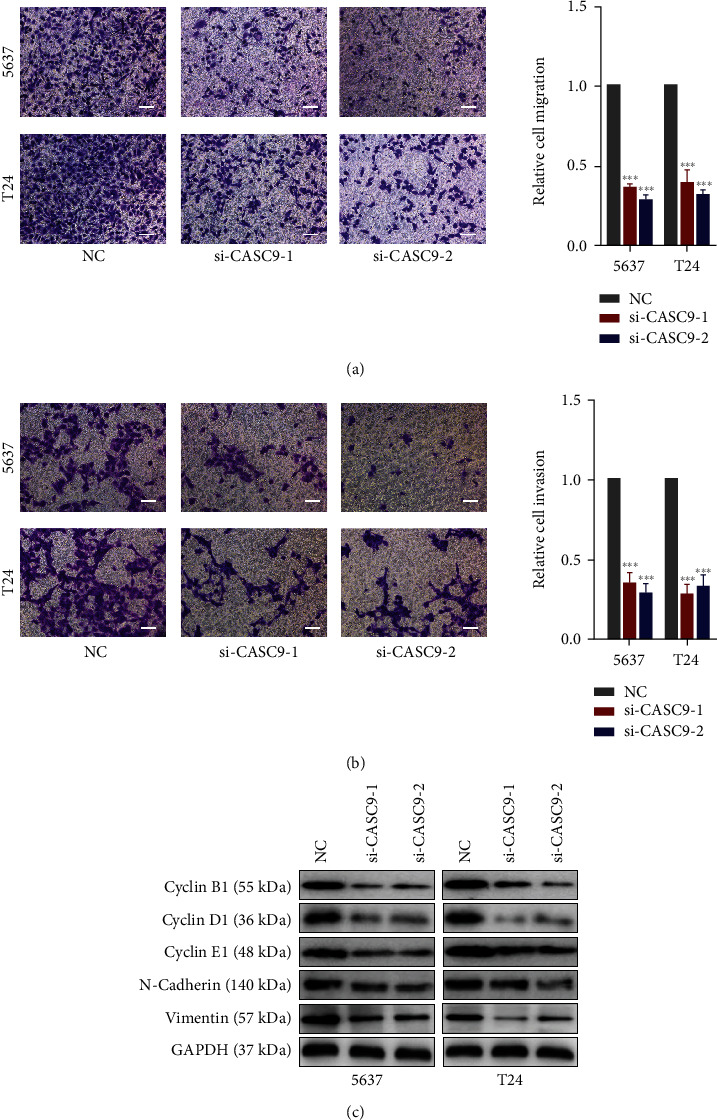
CASC9 promotes BC cells metastasis in vitro. (a) Transwell migration assays of 5637 and T24 cells transfected with CASC9 siRNAs. (b) Transwell invasion assays of 5637 and T24 cells transfected with CASC9 siRNAs. (c) The expression levels of cylcin B1, cylcin D1, cylcin E1, N-cadherin, and vimentin in both 5637 and T24 cells transfected with CASC9 siRNAs were detected by Western blot assay. Scar bar = 50 *μ*m, ^∗^*P* < 0.05, ^∗∗^*P* < 0.01, and ^∗∗∗^*P* < 0.001.

**Figure 5:**
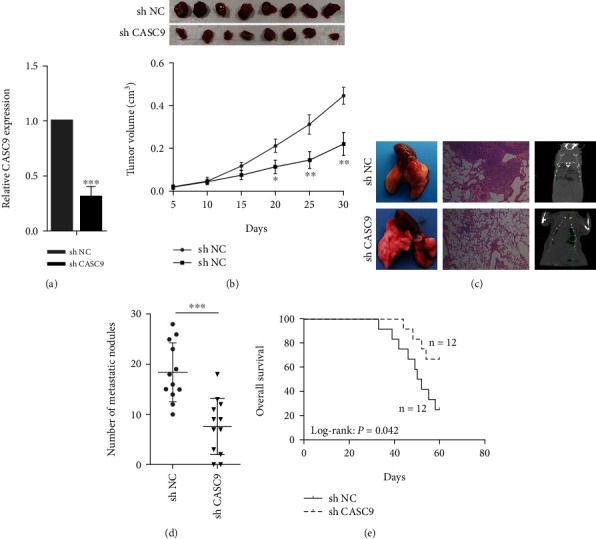
CASC9 promotes BC cells growth and metastasis in vivo. (a) The CASC9 expression was detected in sh-CASC9 stably transfected 5637 by qRT-PCR. (b) The CASC9 stably knock down group or the negative control was used for tumorigenesis assay. One month later, the mice were euthanized, and the tumor nodules were harvested. Tumor growth curves are shown. (c) Representative images of lungs (left) from mice after tail vein injections with stably transfected sh-CASC9 and sh-NC cells. Representative images of H&E staining (middle) and computerized tomography (CT) scan (right). (d) Quantitative analysis of metastasis foci in corresponding groups. (e) Survival analysis of mice after tail vein injections with stably transfected sh-CASC9 and sh-NC cells. ^∗^*P* < 0.05, ^∗∗^*P* < 0.01, and ^∗∗∗^*P* < 0.001.

The role of CASC9 in BCa cells was evaluated through a series of experiments. CCK-8 experiments showed that the proliferation ability of BCa cancer cells was significantly reduced after CASC9 was silenced (Figure 3(a)). Colony formation assays revealed that the ability of BCa cells to form colonies decreased significantly after CASC9 was silenced (Figure 3(b)). The results of EdU experiments indicated that after the expression of CASC9 was inhibited, the proliferation ability of BCa cells was also significantly inhibited, and the positive rate of EdU in the siRNA group was significantly lower than that in the control group (Figure 3(c)). Transwell migration and invasion experiments showed that after CASC9 was knocked down, the migration and invasion ability of BCa cells was significantly reduced (Figures 4(a) and 4(b)). In addition, the results of Western blot experiment displayed that after CASC9 was silenced, the expression levels of the proteins cyclin B1, cyclin D1, and cyclin E1, which reflect the cell proliferation ability and the markers of the epithelial to mesenchymal transitions (EMTs), vimentin, and N-cadherin, were significantly decreased (Figure 4(c)). To investigate the role of CASC9 on BCa in vivo, we constructed a stably transfected 5637 cell line (sh CASC9 5637) with low expression of CASC9 (Figure 5(a)). The results of the subcutaneous xenograft model in nude mice revealed that the volume of the transplanted tumors in the sh CASC9 group was significantly smaller than that in the sh NC group (Figure 5(b)). The results of tail vein injection lung metastasis models showed that compared with the control group, the number of lung metastasis nodules in mice injected with sh CASC9 cells was significantly reduced (Figures 5(c) and 5(d)). The results of the survival analysis indicated that the survival time of the mice in the sh CASC9 group was longer than that of the control group, and the mortality rate was also lower (Figure 5(e)). The above results indicate that silencing CASC9 weakens the growth and metastasis ability of BCa cells both in vitro and in vivo.

### 3.4. CASC9 Acts as ceRNA to Regulate the Expression of TK1 in Bladder Cancer

**Figure 6:**
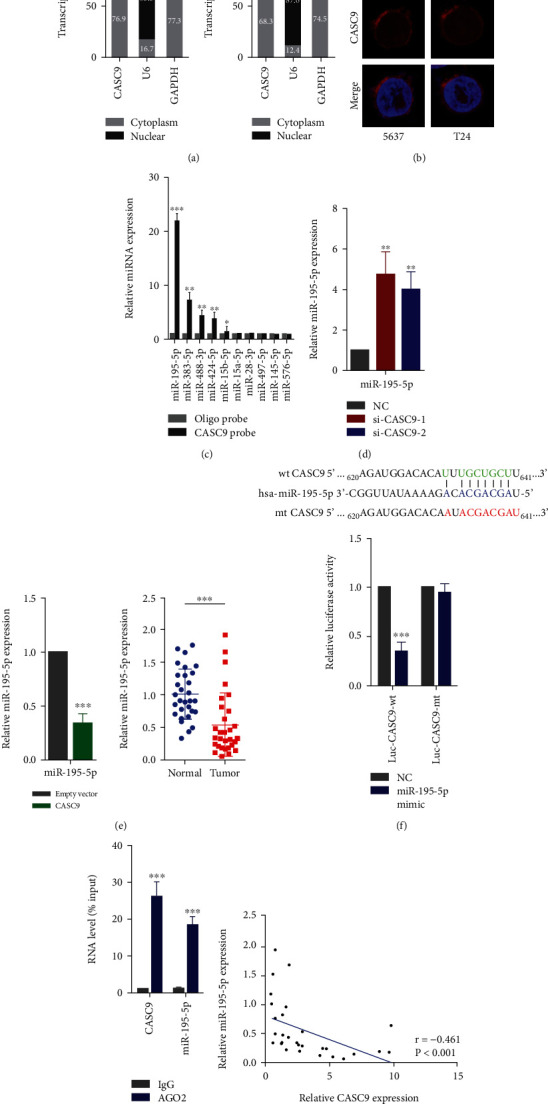
CASC9 directly interacts with miR-195-5p. (a) Relative CASC9 expression levels in the nuclear and cytoplasm fractions of 5637 and T24 cells. Nuclear controls: U6, cytosolic controls: GAPDH. (b) FISH was used to detect CASC9 localization in 5637 and T24 cells. Red: CASC9; blue: DAPI. (c) RNA pull-down assays were used to examine the association of CASC9 and potential target microRNAs. (d) The MiR-195-5p expression was examined in 5637 cells transfected with CASC9 siRNAs (left) or overexpression vectors (right). (e) The MiR-195-5p expression was analyzed by qRT-PCR in BC and adjacent nontumor tissues (*n* = 30). (f) Luciferase reporter assays were used to determine the interacting activity between miR-195-5p and CASC9. (f) RIP assays with an anti-Ago2 antibody to assess endogenous Ago2 binding RNAs and IgG was used as the negative control. The levels of CASC9 and miR-195-5p were determined by qRT–PCR and presented as fold enrichment in Ago2 relative to input. (g) The correlation between CASC9 and miR-195-5p was analyzed in 30 paired BC samples (*r* = 0.−461, *P* < 0.001). ^∗^*P* < 0.05, ^∗∗^*P* < 0.01, and ^∗∗∗^*P* < 0.001.

**Figure 7:**
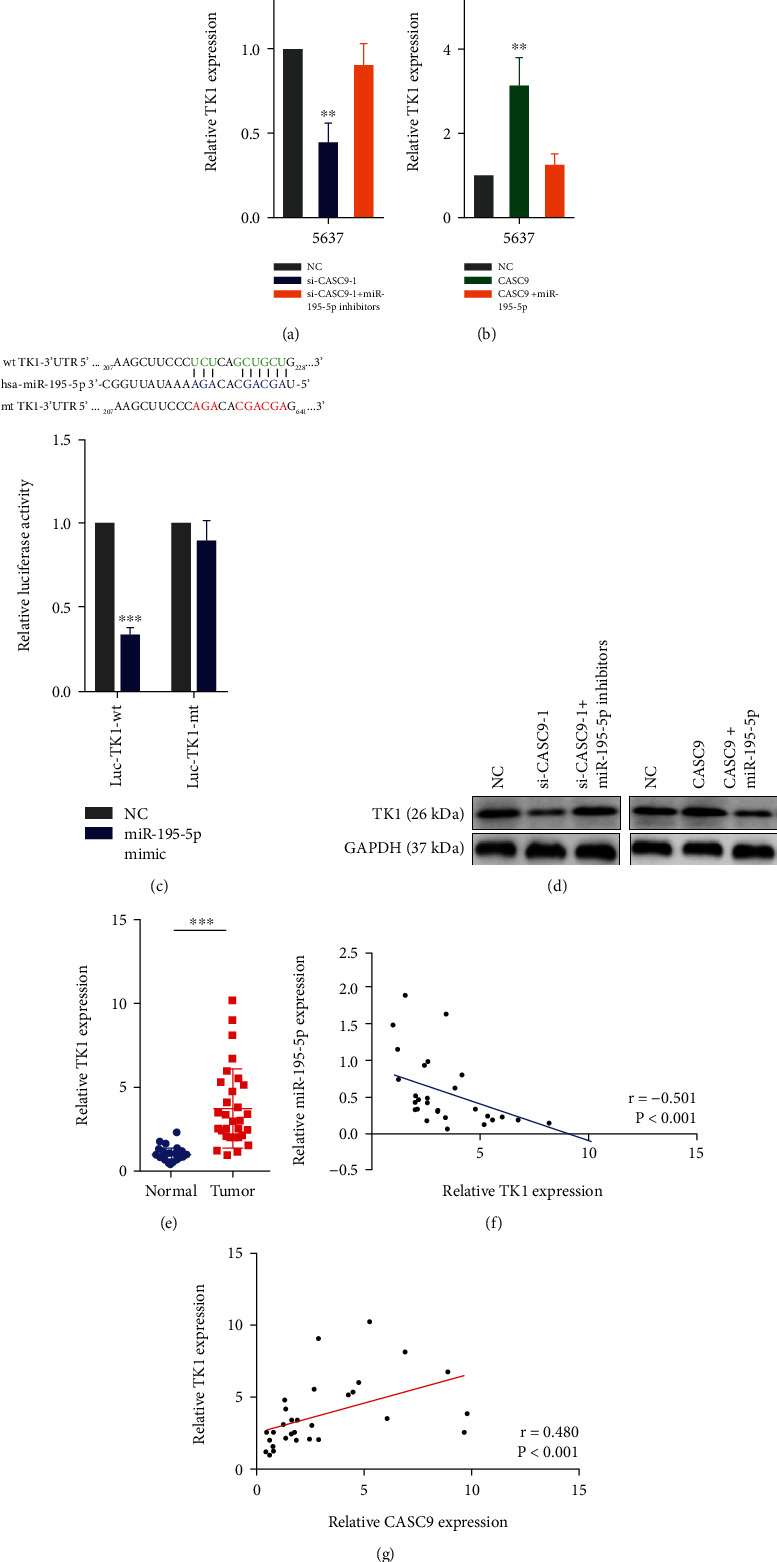
CASC9 functions as ceRNA TK1 in BC cells. (a) The TK1 expression was detected by qPCR in 5637cells transfected with CASC9 siRNAs or cotransfected with CASC9 siRNAs and miR-195-5p inhibitors. (b) The TK1 expression was detected by qPCR in 5637cells transfected with CASC9 vectors or cotransfected with CASC9 vectors and miR-195-5p mimics. (c) Dual luciferase reporter assays were used to determine the miR-195-5p binding on the 3′UTR of TK1. (d) The TK1 expression was detected by Western blot in 5637cells with indicated treatment. (e) QRT-PCR analysis of the TK1 expression in 30 pairs of BC and corresponding adjacent normal tissues. (f) Correlation analysis between TK1 and miR-195-5p in 30 paired BC samples (*r* = 0.−501, *P* < 0.001). (g) Correlation analysis between TK1 and CASC9 in 30 paired BC samples (*r* = 0.480, *P* < 0.001). ^∗∗^*P* < 0.01 and ^∗∗∗^*P* < 0.001.

Then, we investigated the molecular mechanism by which CASC9 played a carcinogenic role in BCa. The results of separation of nuclear and cytoplasmic RNA indicated that CASC9 was mainly located in the cytoplasm (Figure 6(a)). The FISH experiments also confirmed this result (Figure 6(b)). A lncRNA localizes in the cytoplasm can function as ceRNA to regulate the expression of target genes. We used the ENCORI database to predict the microRNAs potentially combined with CASC9. To identify the microRNAs that bind to CASC9, we conducted RNA pull down experiments with biotin-labeled CASC9. The results showed that miR-195-5p, miR-383-5p, miR-488-3p, and miR-424-5p could directly bind to CASC9, and miR-195-5p had the largest binding capacity (Figure 6(c)). So, we selected miR-195-5p for further research. The expression of miR-195-5p was significantly increased after CASC9 was knocked down in 5637 cells. On the contrary, after CASC9 was overexpressed in 5637 cells, the expression of miR-195-5p decreased significantly (Figure 6(d)). The PCR analysis showed that the expression of miR-195-5p in BCa tissue was significantly lower than that in adjacent tissues (Figure 6(e)). In addition, we constructed a wild-type (wt CASC9) and a mutant (mt CASC9) luciferase reporter based on the possible binding sites of miR-195-5p and CASC9. The results of the luciferase reporter assays revealed that the overexpression of miR-195-5p could cause the luciferase activity of the wild-type reporter to decrease but had no effect on the luciferase activity of the mutant luciferase reporter (Figure 6(f)). The RIP experiments also showed that both CASC9 and miR-195-5p can directly bind to AGO2 protein (Figure 6(g)). Besides, correlation analysis revealed a positive correlation between the expression of CASC9 and miR-195-5p (Figure 6(h)). We then used bioinformatic tools and found that thymidine kinase 1 (TK1) was a potential target gene of miR-195-5p. In line with our speculation, when CASC9 was silenced in BCa cells, the expression of TK1 was significantly reduced. When CASC9 was silenced while inhibiting the expression of miR-195-5p, the decreased TK1 expression would be rescued (Figure 7(a)). Conversely, when CASC9 was overexpressed, the expression of TK1 was significantly increased, but when both CASC9 and miR-195-5p was overexpressed, the increase of TK1 was suppressed (Figure 7(b)). To verify that miR-195-5p can directly regulate TK1, we constructed the dual luciferase reporters (Luc-TK1-wt and Luc-TK1-mt) based on the predicted binding sites of miR-195-5p and 3′-UTR of TK1. The experimental results showed that miR-195-5p could directly bind to 3′-UTR of TK1 based on the predicted binding sites (Figure 7(c)). Similarly, the results of Western blot show indicated that the regulation of TK1 by CASC9 was consistent at the protein level and RNA level (Figure 7(d)). In addition, PCR results showed that TK1 was significantly overexpressed in BCa tissues (Figure 7(e)). Expression correlation analysis revealed that in BCa, the expression of TK1 was negatively correlated with miR-195-5p and positively correlated with the expression of CASC9 (Figures 7(f) and 7(g)). These results indicate that CASC9 acts as a ceRNA to regulate TK1 expression in BCa.

### 3.5. CASC9 Exerts an Oncogene Effect by Regulating TK1 in Bladder Cancer

**Figure 8:**
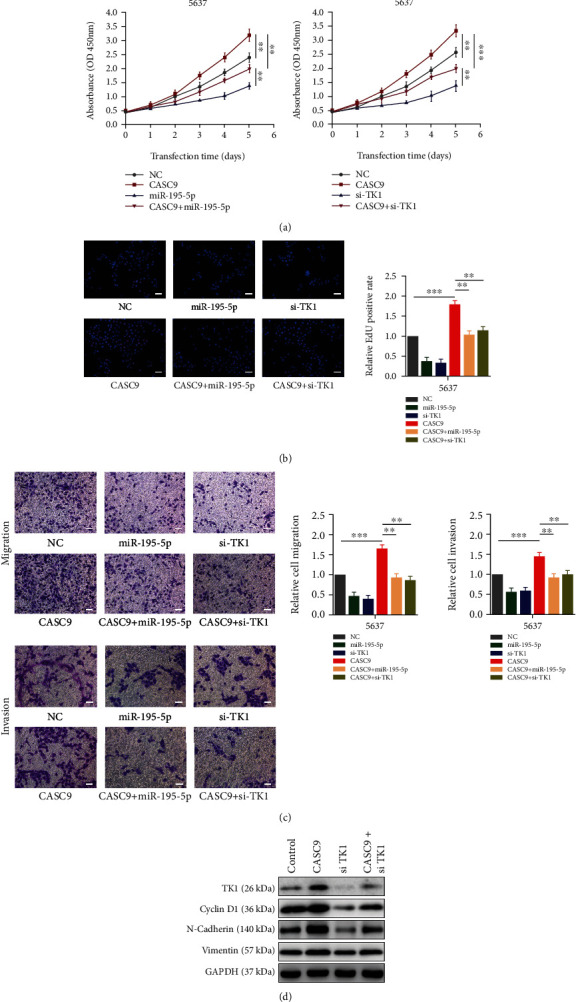
CASC9 promotes BC progression in part by regulating TK1 expression. (a) CCK-8 assays were used to determine viability of 5637 cells with indicated treatment. (b) EdU assays were conducted to determine proliferation rate of 5637 cells with indicated treatment. (c) Transwell assays were performed to determine migration and invasion ability of 5637 cells with indicated treatment. (d) The expression of TK1, cylcin D1, N-cadherin, and vimentin was detected by Western blot in 5637 cells with indicated treatment. Scar bar = 50 *μ*m. ^∗^*P* < 0.05, ^∗∗^*P* < 0.01, and ^∗∗∗^*P* < 0.001.

We further explored whether the role of CASC9 in promoting the growth and metastasis of BCa depends on TK1. CCK8 assay showed that the overexpression of CASC9 promoted the growth of BCa cells. When the overexpression of CASC9 was accompanied by the overexpression of miR-195-5p or inhibition of the TK1 expression, the growth acceleration caused by the overexpression of CASC9 would be weakened (Figure 8(a)). EdU experiments revealed that the overexpression of CASC9 can promote the proliferation of BCa cells. When CASC9 was overexpressed with miR-195-5p overexpression or TK1 inhibition, the increase in proliferation rate caused by the overexpression of CASC9 would be weakened (Figure 8(b)). In addition, the results of transwell assays showed that the overexpression of CASC9 would increase the migration and invasion ability of BCa cells, but the overexpression of miR-195-5p or inhibition of TK1 at the same time of overexpression of CASC9 would impair the increased migration and invasion ability (Figure 8(c)). Besides, Western blot results indicated that the overexpression of CASC9 would cause the elevation of TK1, cyclinD1, vimentin, and N-cadherin, and if the overexpression of CASC9 was accompanied by inhibition of TK1, the elevation trend of these proteins would be eliminated (Figure 8(d)). These results demonstrated that CASC9 promoted the progression of BCa by regulating the expression of TK1.

## 4. Discussion

Increasing evidences have shown that lncRNAs function as key regulators of a variety of malignancies [11–13]. In our work, we identified a number of lncRNAs that were dysregulated in human BCa tissues and found that CASC9 was upregulated in human BCa tissues compared with corresponding nontumor tissues. CASC9 has been reported to be overexpressed in papillary thyroid cancer [14] and colorectal cancer [15]. In our study, lncRNACASC9 was markedly overexpressed in BCa tissues and closely associated with poor OS.

Many transcription factors are reported to be aberrantly expressed in cancer cells, and several of them can induce overexpression of lncRNAs [16–18]. To determine the reason for high CASC9 expression in BCa cells, JASPAR software was used to predict transcription factors that might bind to the CASC9 promoter region, and CHIP and luciferase reporter assays were performed and revealed that NF-*κ*B could bind to the promoter of CASC9 and induce upregulation of CASC9.

A number of previous reports showed that lncRNAs play critical roles in modulating the malignant phenotypes of cancer cells [19–21]. Various in vitro and in vivo assays in our work showed that CASC9 positively modulates proliferation and metastasis of BCa cells. These data indicated that lncRNACASC9 may serve as an oncogene to facilitate tumorigenesis of BCa.

LncRNAs can guide and recruit transcription factors, DNA or histone protein modification enzymes to specific genomic loci, leading to activation of oncogenes or inactivation of tumor suppressors [22]. Additionally, numerous lncRNAs enriched in the cytoplasm and participate in cellular biological processes via regulating mRNA or stability functioning as ceRNA [23]. Here, our RNA FISH and subcellular fractionation location assays indicated that CASC9 was mainly located in the cell cytoplasm in human BCa cells. lncRNAs have been reported to influence mRNA levels acting as ceRNAs, and the ceRNA model has been shown to play key roles in tumorigenesis.

For example, linc00426 may act as a ceRNA, which effectively suppresses the expression of miR-455-5p, thereby modulating the derepression of UBE2V1, a target gene of miR-455-5p in lung adenocarcinoma [24]. In our work, we tried to prove whether CASC9 works as a ceRNA in BCa. Firstly, target-binding sites for miR-195-5p were identified in CASC9 using the ENCORI database. The results of Luciferase reporter and AGO2-RIP assays verified the competitive relationship between CASC9 and miR-195-5p. In previous studies, miR-195-5p has been identified as a tumor suppressor in other carcinomas [25, 26]. In our study, we confirmed that CASC9 competitively binds to miR-195-5p and downregulates it in BCa cells. Additionally, miR-195-5p inversely correlated with CASC9 in human BCa tissues.

In addition, TK1 was verified as a target gene of miR-195-5p and has been identified as a oncogene in other malignancies [27, 28]. TK1 was detected to be positively modulated by CASC9. To investigate whether CASC9 facilitates the malignant progression of BCa via sponging miR-195-5p, serials of rescue assays were performed and found that the promotion of CASC9 in BCa could be weakened by miR-195-5p and TK1 knockdown. Based on the above results, we concluded that CASC9 functioned as a ceRNA to regulate the TK1 expression through competition for miR-195-5p.

In summary, we characterized the expression profile of lncRNS in BCa and found that CASC9 could be a prognostic marker and serve as an oncogenic lncRNA in BCa. Its effects on cell proliferation and metastasis indicate that it exerted oncogenic property in BCa tumorigenesis. miR-195-5p directly targeted CASC9 and inhibited CASC9 expression and function, while CASC9 acts as a molecular sponge for miR-195-5p and regulate its target gene TK1. This reciprocal repression of miR-195-5p and CASC9 may highlight the importance role of RNA-RNA interaction and clarify the mechanism underlying tumor progression, including tumor growth, migration, invasion, and metastasis.

## Data Availability

The data used and analyzed during our study are available from the corresponding author on reasonable request.
